# Inverted Papilloma Originating Primarily from the Nasolacrimal Duct: A Case Report and Review of the Pertinent Literature

**DOI:** 10.1155/2015/123694

**Published:** 2015-11-16

**Authors:** Hussein Z. Walijee, Sandeep Berry, Stuart Quine, Carol Lane, Daniel S. Morris, Benedict Bowman

**Affiliations:** ^1^Department of Otorhinolaryngology, Royal Glamorgan Hospital, Ynysmaerdy, Llantrisant CF72 8XR, UK; ^2^Department of Otorhinolaryngology, University Hospital of Wales, Heath Park, Cardiff CF14 4XW, UK; ^3^Department of Ophthalmology, University Hospital of Wales, Heath Park, Cardiff CF14 4XW, UK; ^4^Department of Cellular Pathology, University Hospital of Wales, Heath Park, Cardiff CF14 4XW, UK

## Abstract

*Introduction.* Inverted papilloma (IP) is an uncommon, benign yet aggressive neoplasm characterised by high recurrence rates and tendency towards malignant transformation. The majority of IP cases originate in the ethmoid region, lateral wall of the nasal fossa, and maxillary sinus. The authors report a case of an IP originating primarily from the nasolacrimal duct (NLD).* Case.* A 69-year-old Caucasian gentleman presented with a lump in his right medial canthal region, epiphora, and discharge bilaterally. Radiological investigation revealed a well-defined, heterogeneous mass within the proximal NLD eroding the bony canal, protruding into the middle meatus and into the right orbit. The tumour was excised en bloc utilizing a combined external and endoscopic approach based on its location. Histology revealed hyperplastic ribbons of basement membrane-enclosed epithelium growing endophytically into the underlying stroma with no evidence of invasive malignancy. The patient made an uneventful recovery with unchanged visual acuity and normal extraocular movements.* Conclusion.* The case demonstrates variability within the sinonasal tract that IP can develop and the individuality of each case necessitating tailored operative techniques for complete excision whilst minimising recurrence rates. We also present a combined endoscopic approach for the en bloc resection of a NLD IP with no clinical recurrence at 15-month follow-up.

## 1. Introduction

Inverted papilloma (IP) is an uncommon, benign yet aggressive epithelial neoplasm, characterised by a high recurrence rate and its propensity to turn malignant. IP originates from the mucosal epithelial lining of the sinonasal tract which is in continuity with the nasolacrimal duct (NLD) and lacrimal sac, with the majority of cases originating in the ethmoid region, lateral wall of the nasal fossa, and maxillary sinus [1, 2]. Though infrequently, the NLD can be involved secondarily due to local extension from the paranasal sinuses or lacrimal sac [3]. The authors report a case of an IP originating primarily from the NLD.

## 2. Clinical History

A 69-year-old gentleman presented to the ophthalmology outpatient department with a varying lump in his right medial canthal region for two years. The patient reported chronic epiphora and discharge bilaterally, denying bloody tearing, pain, or diplopia. A nontender 1.5 cm smooth dome-shaped mass was palpable below the level of the right medial canthal ligament. Syringing revealed 90% flow and 10% reflux following dilatation of the puncta with no mucopurulent regurgitation. Visual acuity was 6/9 and 6/6 in the right and left eyes, respectively, without correction and there was no relative afferent pupillary defect or proptosis. The patient thirty years previously had conjunctival papilloma excised from the right eye with no extension into the medial canthus. He is otherwise fit and well.

**Figure 1:**
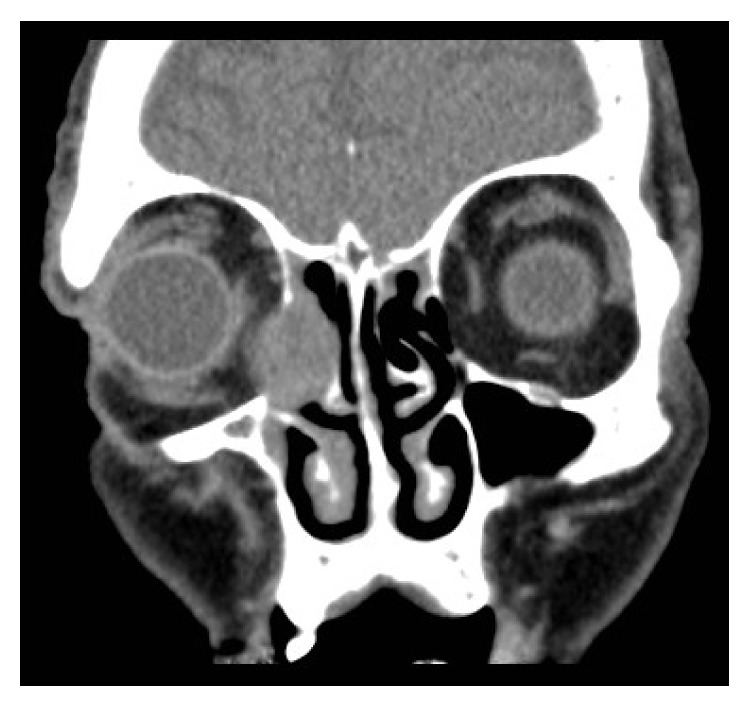
CT orbits: coronal section showing a well-defined heterogeneous lesion protruding into the middle meatus and right orbit.

**Figure 2:**
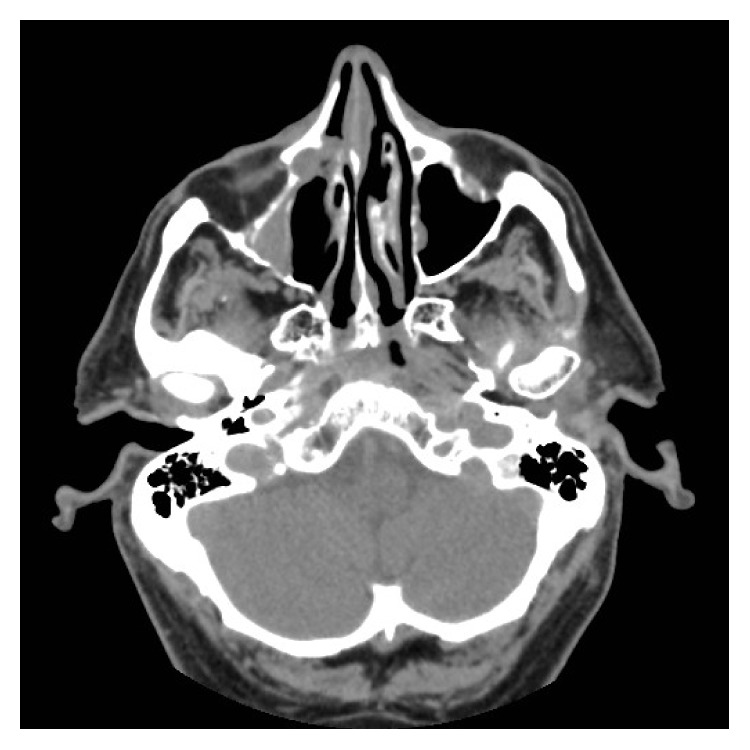
CT sinuses: axial section showing the lesion within the right nasolacrimal duct with evidence of bony remodelling.

Computed Tomography (CT) of the orbits revealed a 2 cm round well-defined, heterogeneously enhancing soft tissue mass within the proximal right NLD causing pressure erosion and thinning of the bony canal, protruding medially into the middle meatus and laterally into the right orbit (Figures 1 and 2).

**Figure 3:**
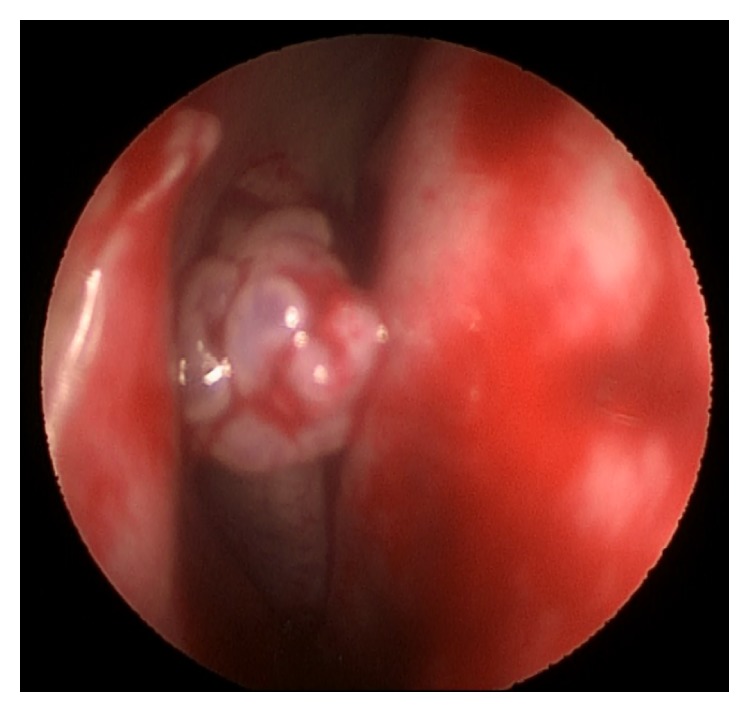
Endoscopic view demonstrating a pale, polypoid-like mass, covered by a papillary surface, protruding from the middle meatus.

Magnetic Resonance Imaging (MRI) of the head confirmed a mass arising from the proximal NLD, mildly hypointense on T2 weighted images, isointense on T1 weighted images, and avidly enhancing on postcontrast images eroding the anterior lamina papyracea and bulging into the medial aspect of the right orbit. Right nasal bones were eroded with the mass extending into the nasal cavity to lie in the middle meatus anteriorly (Figure 3).

An endoscopic biopsy under general anaesthesia revealed a benign squamous papilloma of the lacrimal duct with no evidence of dysplasia or malignancy.

Following discussion at the Neuroradiology and Head and Neck Multidisciplinary meetings, complete resection as a joint case between Otorhinolaryngology, Oral and Maxillofacial Surgery, and Ophthalmology was planned.

A lateral rhinotomy approach was utilized to gain access to the tumour, followed by dissection along the medial wall of orbit and around tumour. The tumour was dissected free from its remodelled lacrimal fossa and the common canaliculus divided laterally to the tumour. The anterior buttress of the frontal process of maxilla overlying the remodelled NLD was drilled away. Right inferior turbinate, middle turbinate, and its posterior stump were removed endoscopically to expose the ostium. The tumour was thereby excised en bloc using a combined external and endoscopic approach. The lacrimal drainage pathway was not reconstructed on this occasion.

**Figure 4:**
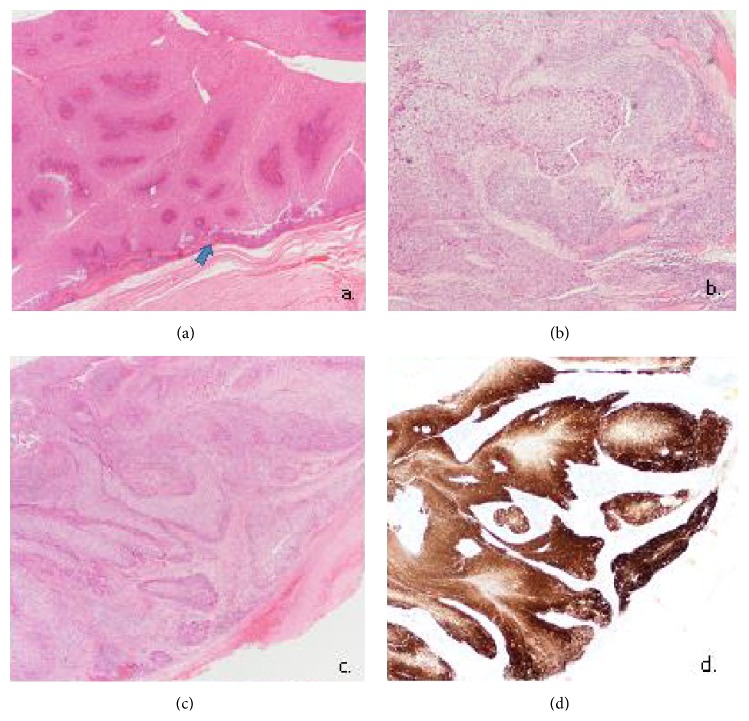
(a) Hyperplastic squamous epithelium growing endophytically. The inverted papilloma is seen to arise from nasolacrimal duct epithelium (arrow). Haematoxylin and Eosin, 20x. (b) Foci of atypia amounting to moderate dysplasia. No invasive malignancy is seen. Haematoxylin and Eosin, 40x. (c) Inverted pattern of growth. Haematoxylin and Eosin, 20x. (d) Immunohistochemistry for P16 showing block positivity. P16 IHC Syntech antibody, 20x.

Histological examination revealed hyperplastic ribbons of basement membrane-enclosed epithelium growing endophytically into the underlying stroma (Figures 4(a) and 4(c)). The epithelium is multilayered, 5–30 cell layers thick, and formed of squamous epithelial cells admixed with scattered mucocytes. Multiple foci of atypia, interpreted as moderate dysplasia, were reported with no overall evidence of invasive malignancy (Figure 4(b)). The patient was therefore a Krouse Stage 3 on the Krouse Staging System [4]. Immunohistochemistry was positive for p16 (Figure 4(d)).

The patient recovered with unchanged visual acuity and normal extraocular movements following surgery with no evidence of recurrence clinically at 15-month follow-up.

## 3. Discussion

The Schneiderian papilloma was first described in 1854 by Ward and Billroth [5]. In 1938, Ringertz [6] coined the term “inverted papilloma” based on the histopathological appearance of epithelium inverting into the underlying stroma. Generally, IP accounts for approximately 0.5% to 4% of all surgically resected nasal tumours [2] and 70% of sinonasal papillomas [7]. Incidence of IP ranges from 0.6 to 1.5 cases per 100.000 inhabitants per year [2]. Lesions are commonly unilateral, three times as common in males [8], and have the highest incidence in the sixth and seventh decades of life [9]. Isolated observations in the paediatric age groups have been reported [10]. The rate of malignant transformation mainly into Squamous Cell Carcinoma (SCC) has been quoted in the literature within 5–21% [11–13].

The lacrimal drainage system is an uncommon [3] primary location for IP having been reported in only 21 cases over the last 20 years [1, 14–21]. To our knowledge, this is one of the first reports of an IP originating primarily in the NLD. Hyams [22] suggested that ectopic migration of the Schneiderian membrane during embryogenesis could explain the unusual site of papillomas contiguous with the sinonasal tract.

Lacrimal system papillomas largely present with epiphora and a medial canthal mass if detected early during the course of the disease. In advanced cases, proptosis, nonaxial globe displacement, skin ulceration, and invasion of adjacent structures can occur [7].

Preoperative radiological planning is an important step in determining the extent of the lesion and invasion into adjacent structures. CT and MRI together are the recommended technique for pretreatment staging in IP as they are complementary in the accurate evaluation of disease extension and in differentiating tumour from inflamed mucosa, retained secretions, and granulation tissue [23]. Features suggestive of IP on CT include a unilateral mass with a lobulated pattern [23], bony remodelling, and sclerosis. Erosion of the lamina papyracea, medial sphenoid wing, cribriform plate, or the NLD is seen in advanced cases [24]. The IP itself may contain calcified areas which may appear as areas of hyperdensity. On MRI, they appear heterogeneous with an intermediate signal intensity on T2 weighted images [23].

IP has the ability to destroy adjacent bone even in the absence of malignant transformation; therefore, an early, well-planned wide excision of the lesion, diseased mucosa, and the underlying mucoperiosteum [25, 26] should be performed as primary treatment to reduce recurrence rates [13, 21, 27].

In the last 25 years, treatment strategy for sinonasal IP has evolved due to advancements in endoscopic surgery. Traditionally, medial maxillectomies via lateral rhinotomy or midfacial degloving was considered gold standard [9, 27]. More recently, combined endoscopic-assisted procedures and purely endoscopic procedures [13, 15] have achieved recurrence rates that are comparable if not better than more invasive techniques [1, 26–28].

A review of the literature by Lund et al. [2] found that the recurrence rate for patients treated with a purely endoscopic technique was 14.5% at 37-month follow-up, comparable to the group treated with a lateral rhinotomy and medial maxillectomy with a recurrence rate of 16.7% at 62-month follow-up. Limited resections were reported to have a recurrence rate of 34.4%.

Woodworth et al. [12] describe 24 patients who underwent a combined endoscopic and Caldwell Luc approach with one recurrence (mean disease-free follow-up of 40 months).

Tumour location is an important factor influencing the choice of approach [25]. Sham, 1998 [28], recommends an endoscopic approach, supplemented by targeted extranasal approaches for endoscopically inaccessible tumours, the method employed in the case presented. A large case series of 200 patients treated at the Mount Sinai Medical Centre reported an increased likelihood of requiring an adjunctive extranasal approach in secondary (19/32) and Krouse Stage 4 (10/10) cases [11].

A meta-analysis suggested that piecemeal resection of IP via an endoscopic approach did not compromise tumour control rates compared to en bloc external techniques. The authors advocate careful identification and complete removal of the site of attachment of the lesion [28]. In contrast, Sauter et al. [27] recommend en bloc resection of the lateral nasal wall, ethmoid labyrinth, and medial portion of the maxilla which are potential sites for tumour extension, for successful tumour control.

Adjuvant treatment with radiotherapy could be considered for patients with early or multiple recurrence, advanced stage lesions, aggressive tumours, incompletely resected lesions, lesions with positive margins, and tumours associated with malignancy [29].

Several studies since the early 1980s have postulated the aetiological role of HPV in the development of IP. Syrjänen 2003 [30] found HPV subtypes 6 and 11 in 33.3% of over 1,000 IP in 2003. Interferon and other antiviral agents like intralesional therapy with Cidofovir have been suggested for use in patients with multiple recurrences and advanced disease [29].

Topical interferon alpha-2b therapy was first used successfully in 2002 on a 10-year-old patient presenting with a recurrent lacrimal system papilloma, with no recurrence at 12 months [31]. Woodcock et al. [32] reported using Mitomycin C irrigation effectively as an adjunct immediately following resection of a recurrent IP in a 33-year-old female with no recurrence at 18-month follow-up.

Long-term follow-up for IP is essential. Most recurrences are seen within 9 months but can occur many years later. An analysis of 3058 cases by Mirza et al. [33] found a 3.6% prevalence of metachronous carcinoma with a mean interval of 52 months, emphasizing the need for follow-up for at least 5 years.

The case presented demonstrates the variability of location within the sinonasal tract that IP can develop, and the individuality of each case necessitates tailored operative techniques for complete excision whilst minimising recurrence rates. We also present a combined endoscopic approach for the en bloc resection of a NLD IP with no clinical recurrence at 15-month follow-up.
